# A Manual of Medical Jurisprudence

**Published:** 1873-11

**Authors:** 


### Books Reviewed

*A Manual of Medical Jurisprudence.* By Alfred Swaine Taylor, M. D., F. R. S. Second American Edition, edited by John J. Reese, M. D. With illustrations on wood. Philadelphia: Henry C. Lea, 1873. Buffalo: T. Butler & Son.

The favorable reception which has been accorded the former editions of Taylor's Medical Jurisprudence, renders it unnecessary for us to give a critical review of its contents. The present edition is increased by about one hundred pages over the former, and several subjects are introduced not treated in that edition. Most of the notes of the former editors Dr Hartshorne and Mr. Penrose, have been retained, and are distinguished by the initials, H. and P.

The publication of a larger work by the same author entitled the "Principles and Practice of Medical Jurisprudence, has rendered a detailed statement of cases unnecessary in this work; these are therefore omitted and room is given for a more complete consideration of the different subjects introduced.

Two very instructive chapters on evidence and the duties and responsibilities of medical witnesses have been placed at the commencement of the volume, these if studied by the profession and borne in mind when on the witness stand, would prevent many medical men from making inexcusable blunders in their manner of giving testimony.

The volume seems to lack nothing as a Manual of Medical Jurisprudence, and it with its companion volumes on The Principles and Practice of Medical Jurisprudence, which are shortly to be published, should be in the library of every physician and practicing attorney. The work is illustrated by several engravings on wood, and the printing and binding are in every way well done.

